# Age- and sex-dependent retinal degeneration in peripherin-2 mutant mice, a model of central areolar choroidal dystrophy

**DOI:** 10.1038/s41420-026-03263-z

**Published:** 2026-07-23

**Authors:** Enola Missonnier, Lorena Vidal-Gil, Carla Sánchez-Castillo, Estela Tébar-Garcerán, Ceren Sahin, Marina Pastor-Mas, Yoel Hernández, Oksana Kutsyr, Laura Fernández-Sánchez, Victoria Maneu, Nicolás Cuenca, Natalia Martínez-Gil

**Affiliations:** 1 Department of Physiology, Genetics and Microbiology, University of Alicante, Alicante, Spain; 2 Department of Optics, Pharmacology, and Anatomy, University of Alicante, Alicante, Spain; 3 Alicante Institute for Health and Biomedical Research (ISABIAL), Alicante, Spain; 4 Department of Biomedical Sciences, Faculty of Health Sciences, Cardenal Herrera-CEU University, Valencia, Spain; 5 CellSight Ocular Stem Cell and Regeneration Program, Department of Ophthalmology, Sue Anschutz-Rodgers Eye Center, University of Colorado, School of Medicine, Aurora, CO USA

**Keywords:** Retinal diseases, Neurophysiology

## Abstract

Central areolar choroidal dystrophy (CACD) is a progressive macular dystrophy without treatment. Although PRPH2 mutations are the most common cause of disease, the mechanisms driving their phenotypic variability remain poorly understood. Here, a comprehensive characterization of the pathophysiological consequences of p.Arg195Leu mutation in PRPH2 was carried out in a mouse model (Prph2^KI/WT^). For evaluating the retinal degeneration, this study combines the analysis of electroretinographic responses together with a complete study of bulk RNA-seq transcriptomics and the key cellular and molecular pathways using confocal imaging, flow-cytometry and western blotting. Results from this work demonstrate that ageing and sex influence retinal degeneration. In young mutant mice, retinal functional impairment and structural disorganization of photoreceptor outer segments were accompanied by a reduced expression of Prph2 and Rom1 genes, together with the activation of the immune system and complement pathways. Middle-age stages represent a critical transition point where the increase of cell death and epithelial barrier dysfunction mark the beginning of retinal degeneration. These pathological events become evident at 9 months of age, where visual pathways-related genes were deregulated. Functional, cellular and molecular alterations observed in the mutant mice do not affect males and females equally. From the earliest stages of the disease, females exhibited greater and sustained inflammatory activation, mainly promoted by complement system upregulation and increased CD11b immunoreactivity, and potentially mediated by IL-6/STAT3/ERK signaling. Moreover, females had greater functional decline and photoreceptor loss. Together, these results could explain the high inter- and intrafamilial variability observed in CACD patients carrying the same mutation. Our findings identify inflammatory biomarkers accompanied by visual function loss, prior to evident retinal degeneration, and demonstrate that age and sex critically shape disease onset and severity. These insights underscore the necessity of incorporating sex-specific biology and early anti-inflammatory treatment into the development of targeted therapies for PRPH2-related dystrophies.

## Introduction

Central areolar choroidal dystrophy (CACD) is considered a rare disease with a prevalence of 1-9 cases per 100,000 individuals. This inherited retinal disorder is characterized by progressive vision loss triggered by the degeneration of photoreceptors (mainly cones), retinal pigment epithelium (RPE), and choroid in the central macula [1]. Although 17 different mutations in six genes could cause CACD, up to 85% of the mutations described in published studies involve the peripherin-2 (*Prph2*) gene [2]. In Spain, the predominant mutation is p.Arg195Leu (c.584G>T) and exhibits high penetrance within affected families [3, 4].

The p.Arg195Leu mutation affects the conformation of intradiscal domain 2 (ID2) of PRPH2 protein, corresponding to the second extracellular (D2) loop [5, 6]. The importance of this domain resides for its role for PRPH2 oligomerization, including interactions with retinal outer segment membrane protein 1 (ROM1) and for proper disc formation of photoreceptor outer segments [7]. Even more, the fact that this mutation is located at this position, could explain the phenotypic heterogeneity in both the clinical manifestations and in the age of onset of clinical signs described in these patients [8, 9]. Recent studies in large cohorts of patients carrying *Prph2* mutations have revealed a spectrum of phenotypes including CACD, pattern dystrophy, pseudo-Stargardt, and retinitis pigmentosa [3, 4, 6, 9]. The discrepancies between the anatomical changes and functional responses in patients carrying same mutation can be explained considering that rods and cones exhibit divergent susceptibility to the damage due to mutation, resulting in cell type-specific degenerative mechanism [8].

Our previous work has provided a detailed characterization of disease progression in this model, highlighting a staged progression of the retinal degeneration [10]. A progressive decline in retinal function was observed in mutant animals, with significantly reduced visual acuity detectable from 6 months of age in *Prph2*^KI/WT^ mice. This functional impairment is accompanied by a gradual decrease in retinal thickness. At the structural level, alterations of the photoreceptor outer segments were reported, together with changes in synaptic connectivity within the outer plexiform layer. In addition, signs of reactive gliosis and microglial activation were observed, suggesting the involvement of neuroinflammatory processes as degeneration progresses. All these signs of degeneration occur earlier and with greater severity in homozygous mutants, indicating a gene dosage effect on disease progression [10].

Together, these findings define a progressive cascade from early functional deficits to structural disorganization and retinal remodeling. This temporal framework is essential for interpreting the molecular changes investigated in the present study, as it allows us to distinguish early events potentially involved in disease initiation from later alterations associated with photoreceptor loss.

Therefore, the aim of this work is to conduct a comprehensive study of the retinal pathophysiological mechanisms of the p.Arg195Leu mutation. Using the mouse model generated by our group that recapitulates the progression of the retinal degeneration in CACD patients [10], we aimed to study the resulting retinal phenotypes along the disease. Based on a transcriptomic analysis, we characterized age- and sex-dependent changes, and the molecular pathways potentially involved in the degenerative process, that could promote the inter- and intra-familiar phenotypic heterogeneity of the disease. Herein, we provide new insights of how same *Prph2* mutation differentially affects according to biological factors such as age and sex, as well as the influence of retinal homeostasis. Considering there is no cure for these retinal dystrophies, our results could contribute to the development of therapeutic strategies not only for this mutation but also for those that affect the D2 loop of PRPH2.

## Results

### Functional and structural retinal degeneration is accompanied by age- and sex- dependent molecular changes

**Fig. 1:**
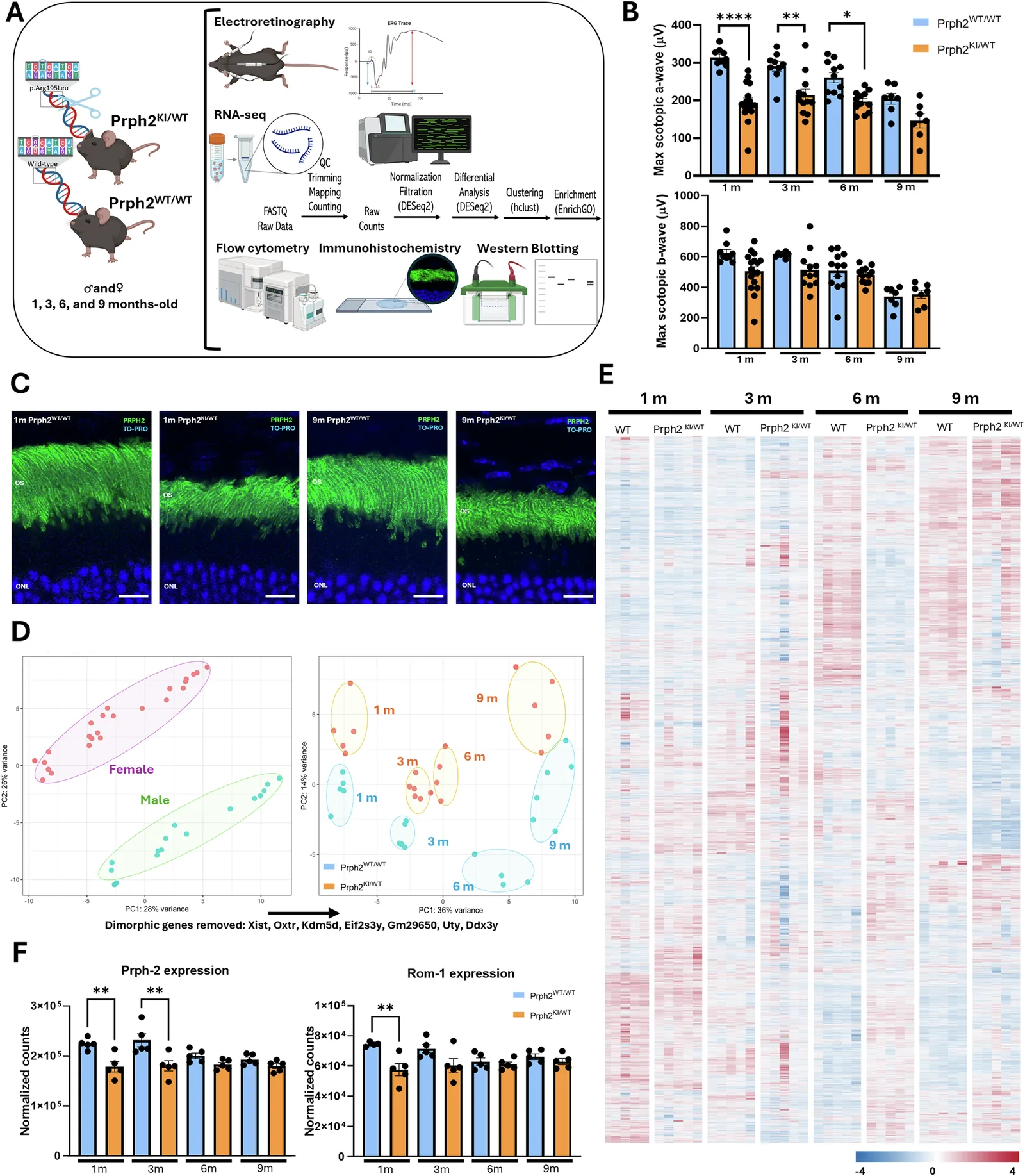
Comprehensive analysis of Prph2 mice degeneration along age. **A** Schematic overview of the experimental workflow used in this study. Created in BioRender. Missonnier Enola. (2025). BioRender.com. **B** Bar graph showing the maximum amplitudes (µV) of the scotopic a-wave and b-wave recorded at the highest light intensity used (1 log cd s/m^2^) in ERGs along age in both experimental groups. For the a-wave analysis, *n* = 38 in WT group (1 m, *n* = 9; 3 m, *n* = 9; 6 m, *n* = 11; and 9 m, *n* = 7), and *n* = 48 in *Prph2*^KI/WT^ group (1 m, *n* = 17; 3 m, *n* = 12; 6 m, *n* = 12; and 9 m, *n* = 7). For the b-wave analysis, *n* = 35 in WT group (1 m, *n* = 9; 3 m, *n* = 7; 6 m, *n* = 12; and 9 m, *n* = 7), and *n* = 48 in *Prph2*^KI/WT^ group (1 m, *n* = 17; 3 m, *n* = 12; 6 m, *n* = 12; and 9 m, *n* = 7). **C** Representative immunofluorescence pictures of retinal cryosections from wild-type (WT) and Prph2 mutant mice (*Prph2*^KI/WT^) at 1 and 9 month-age. Note, in green, the disorganized outer segments (OS) in *Prph2*^KI/WT^ mice compared with WT. Scale bars 10 µm. **D** Principal Component Analysis (PCA) of RNA-seq data illustrating the variance in gene expression between experimental groups, before (left) and after (right) removing dimorphic genes. **E** Heatmap displaying the overall gene expression patterns, with normalized expression (z-score) of genes across all retinal samples, including WT and *Prph2*^KI/WT^ at 1, 3, 6, and 9 months of age. Columns represent individual samples, and rows correspond to genes. The color scale ranges from low express genes in blue to high expression in red. **F** Bar graph showing the Prph2 (left) and Rom1 (right) gene expression Trimmed Mean of M-value (TMM) along age in both experimental groups analyzed by RNA-seq. Transcriptomic analyses (panels **D**–**F**) were performed using the same set of animals with *n* = 20 per experimental group (*n* = 5 per age group, 4 age groups: 1, 3, 6, and 9 months). Values are expressed as mean ± SEM, *p-value < 0.05; **p-value < 0.01; ***p-value < 0.0001.

To characterize the retinal degeneration in *Prph2*^KI/WT^ mice, as the experimental pipeline shows (Fig. 1A), a comprehensive analysis was carried out evaluating the retinal degeneration ranging from physiological function to molecular-level alterations. This study includes not only the analysis of retinal function and outer segment morphology, but also the transcriptomic profile and cellular signaling pathways activated in the retina of mutant mice compared with their wild-type counterparts.

The ERG data reported a significant and progressive age-dependent degeneration of retinal function in *Prph2*^KI/WT^ mouse model compared to *Prph2*^WT/WT^ (Fig. 1B). Maximum scotopic a-wave values significantly decreased in the knock-in mice from 1 month to 6 months of age compared with the corresponding control groups. Moreover, maximum scotopic b-wave values suggested a decreasing trend in the ERG responses at 1 and 3 months, although these results were not statistically significant.

Consistent with the functional results, the morphology of the photoreceptor outer segments (OS) was also altered at just one month of age in mutant mice. The PRPH2 immunostaining (green) revealed an evident altered structural organization at OS level in *Prph2*^KI/WT^ mice, which becomes more evident at 9 months of age (Fig. 1C). Moreover, these changes were not observed in the control group.

After bulk RNA sequencing, global data structure was first investigated by Principal Component Analysis (PCA) (Fig. 1D). The analysis revealed a marked influence of sex on retinal gene expression. Excluding the dimorphic genes, PCA plot reveals a progressive transcriptomic divergence along age and phenotype showing 2 different profiles between *Prph2*^KI/WT^ and *Prph2*^WT/WT^ mice models. After removing the dimorphic genes, groups are consistent in all experimental replicates. While in *Prph2*^WT/WT^ samples there is a clear difference in variance across the studied ages (1 m, 3 m, 6 m and 9 m), the *Prph2*^KI/WT^ group exhibits a thin variance between 3 and 6 months old. The exploratory heatmap of the gene expression profile from all retinas included in this study shows the inter-group differences at all studied ages. In addition, it highlights that the most pronounced divergence between control and mutant mice arises at 3 and 6 months of age, indicating these ages as a critical time points for gene expression deregulation in *Prph2*^KI/WT^ mice (Fig. 1E).

To assess whether the p.Arg195Leu mutation in *Prph2* gene directly affects not only its own transcription but also the transcription of the *Rom1* gene, which interacts functionally with PRPH2, the expression of both genes was analyzed across different ages in both groups. As it can be seen in Fig. 1F, *Prph2* expression was significantly reduced at 1 and 3 months of age in *Prph2*^KI/WT^ mice compared to *Prph2*^WTWT^. Importantly, *Rom1* expression also displayed a significant reduction at 1 month in mutant mice in comparison with wild-type animals.

### Enrichment analysis of differentially expressed genes reflects the dynamic molecular events underlying the retinal degenerative process in *Prph2*^KI/WT^ mice

**Fig. 2:**
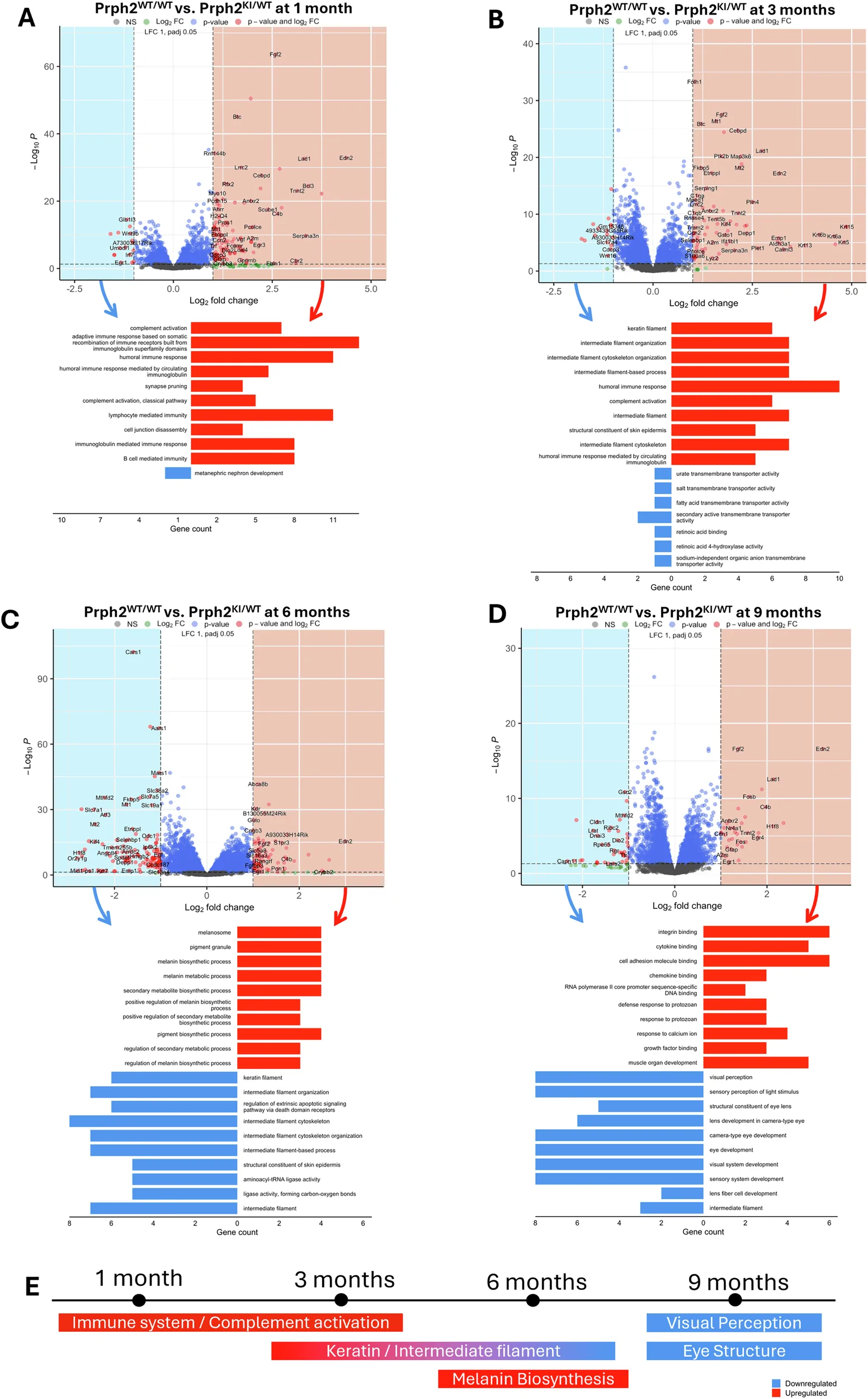
Differential gene expression analysis and enrichment of biological processes across retinal degeneration in Prph2 knock-in mice. **A**–**D** Volcano plots of Differential Expressed Genes (DEGs) in *Prph2*^KI/WT^ compared with *Prph2*^WT/WT^ at 1, 3, 6 and 9 months, respectively. DEGs were defined with log2FC in the range [−1, 1] and adjusted *p*-value (*p*.adj) ≤0.05. Miroir plot (bottom) represent GOterm enrichment analysis of DEGs in *Prph2*^KI/WT^ compared with *Prph2*^WT/WT^ at each time point. **E** Summary of key deregulated pathways throughout the retinal degeneration process in *Prph2*^KI/WT^. Upregulated terms are highlighted in red, and downregulated terms are shown in blue in all graphs. All analyses were performed using the same set of animals with *n* = 20 per experimental group (*n* = 5 per age group, 4 age groups: 1, 3, 6, and 9 months).

Then, we performed a characterization of transcriptomic changes linked to degeneration (Fig. 2). For age-wise differential expression analysis, significant genes were defined as those with |Log2 Fold Change (LFC)| ≥ 1 and adjusted *p*-value (*P*adj) ≤ 0.05. The corresponding volcano plots and GO enrichment analyses at 1, 3, 6, and 9 months-old in both experimental groups are shown respectively in Fig. 2A–D.

The mRNA profile of *Prph2*^KI/WT^ group at 1-month-old displays 132 differentially expressed genes (DEGs) compared to *Prph2*^WT/WT^ mice at same age (Fig. 2A). In total, 116 upregulated genes were identified, being *Fgf2, Btc, Rnf144b, Edn2, Lad1, Lrrc2, Cebpd, Rfx2, Bcl3, Tnnt2* the ten most strongly upregulated. These genes are involved in different immune-related pathways such as “adaptive and humoral responses”, “B-cell immunity”, “complement activation”. In addition, genes with an important role in synaptic remodeling processes like “synapse pruning” and “cell junction disassembly” were also found upregulated. Among the 16 downregulated genes, the top 10 were *Egr1, Irf7, Glb1l3, Wnt9b, Spon2, Depp1, Umodl1, and Bcl2l10* which the enrichment analysis identified only in one pathway “nephron development”, that is unrelated to the tissue studied. The analysis at 3 months reveals 98 DEGs in *Prph2*^KI/WT^ retinas compared to *Prph2*^WT/WT^ ones (Fig. 2B) with a higher proportion of overexpressed genes [82] compared to those showing decreased expression [11]. The upregulated pathways showed again enriched immune-related pathways, including “humoral immune response” and “complement activation”. Furthermore, cytoskeletal remodeling with pathways such as “intermediate filament organization” and “cytoskeleton”, and “keratin filament” appear for the first time with a total of 14 genes involved, such as *Krt15, Krt13, Krt6b, Krt7, Krt6a, Krt4, Krt5*. Conversely, downregulated genes showed enrichment in transmembrane transport, including “fatty acid transporters”. A total of 200 DEGs in *Prph2*^KI/WT^ compared with control group at 6 months of age were reported (Fig. 2C). Of these, 76 genes were upregulated including *End2, Abca8b, and C4b* among others. These upregulated genes are mainly implicated in the melanin biosynthetic process and pigment granules formation. Of the 124 downregulated genes, most of them are involved in the intermediate filament organization of the cytoskeleton and apoptosis regulation. Both 3- and 6-month-olds show a downregulation of intermediate filament organization, with the same pathways affected.

Finally, it is important to note that the difference in the expression of genes implicated in visual pathways was observed only at 9 months of age. Transcriptomic analysis revealed 81 DEGs in *Prph2*^KI/WT^ group compared to control mice (Fig. 2D), with 36 genes upregulated. Among them, *End2, Fgf2, Lad1, Fosb and C4b* stands out. Inside of the 45 downregulated genes *Capn11, Lars2, Ttr, Dnai3, Rpe65, Cldn1, and Lrat* can be found. Pathways like “visual perception”, “light perception of light” and “eye development” were downregulated while upregulated pathways included “integrin binding”, “cell adhesion molecule binding”, and “cytokine binding”. The Fig. 2E summarizes the main dynamic transcriptional changes during retinal degeneration of *Prph2*^KI/WT^ at the studied time points. At early stages of degeneration (1-3 months), the upregulated pathways (red) include the immune system and complement activation as well as keratin synthesis. At later stages, the downregulated pathways (blue) are related to visual perception and eye structure. However, in the mild stage (3-6 months) the analysis showed a transcriptional shift. This is preceded by upregulation of the immune system at 3 months, followed by increased expression of genes related to melanin synthesis, and accompanied by a decrease in remodeling pathways at 6 months.

**Fig. 3:**
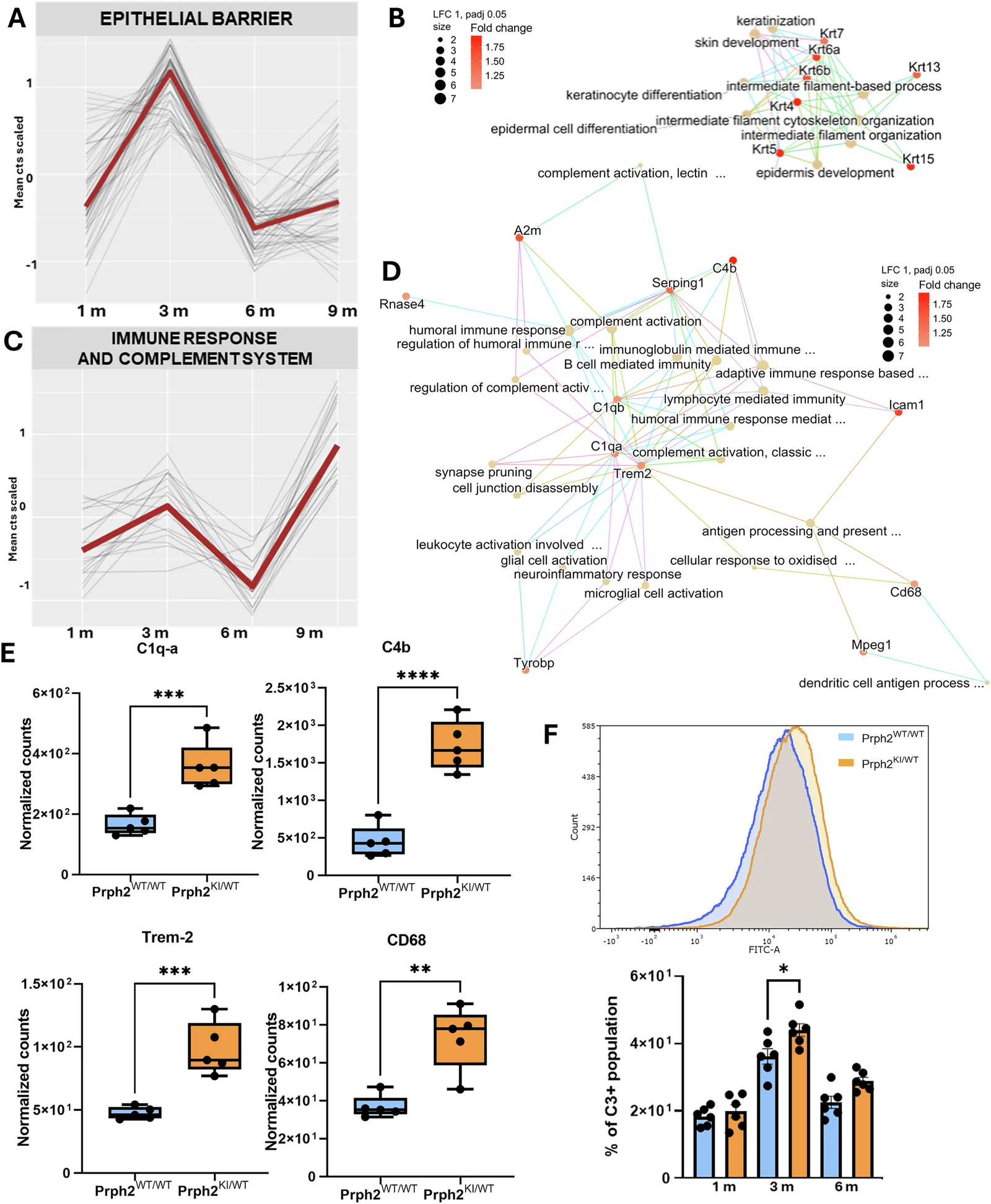
Age-dependent transcriptomic profiles of DEGs engaged in epithelial integrity and immune-complement activation in *Prph2*^KI/WT^. **A** Line plot of the temporal expression profiles gene clusters associated with epithelial barrier function in *Prph2*^KI/WT^. **B** Gene-concept network (Cnetplot) showing DEGs and related pathways clustered in epithelial barrier organization at 3 months of age. **C** Line plot depicts the temporal expression profiles of gene clusters related to immune response and complement system in *Prph2*^KI/WT^. **D** Cnetplot displaying DEGs and related pathways clustered in immune response and complement system at 3 months of age. **E** Box plot of normalized counts (TMM) of immune response and complement system markers overexpressed in *Prph2*^KI/WT^ compared to *Prph2*^WT/WT^ at 3 months of age. Transcriptomic analyses (panels **A**–**E**) were performed using the same set of animals with *n* = 20 per experimental group (*n* = 5 per age group, 4 age groups: 1, 3, 6, and 9 months). **F** Histogram showing C3 immunofluorescence (merged values) in *Prph2*^KI/WT^ and *Prph2*^WT/WT^ animals. Bar graphs represent the percentage of C3 immunoreactive cell population from 1 to 6 months in the two genotypes. *N* = 18 per experimental group (*n* = 6 per age group, 3 age groups: 3, 6, and 9 months). Values are expressed as mean ± SEM *p-value ≤ 0.05; **p-value ≤ 0.01; ***p-value ≤ 0.001; ****p-value ≤ 0.0001.

To explore the temporal dynamics of gene expression, the DEGs identified at each age point were studied across the four established time points. A clustering analysis was performed to evaluate how the gene expression changes over time and to identify potential age-dependent transcriptional patterns. The Fig. 3 shows the clustering of DEGs identified at 3 months in *Prph2*^KI/WT^ mice. The biological pathway enrichment revealed two different clusters of genes that display a similar expression pattern along age. The first one is related to the maintenance of the outer blood–retinal barrier function of the RPE (Fig. 3A) and is characterized by a pronounced expression peak at 3 months. This was driven by the higher expression of keratin genes, including *Krt5, Krt4, Krt6a, Krt6b, Krt7, Krt15*, and *Krt13* at this time point. The Cnetplot in Fig. 3B illustrates the relationship between these genes and their corresponding enriched biological pathways such as “intermediate filament organization”, “intermediate filament cytoskeleton organization”, “intermediate filament-based process”, and “skin development”. Additional enrichment was found in “keratinization”, “keratinocyte differentiation”, and “epidermal cell differentiation” that represent a major contribution of filament remodeling and epidermal development pathways during this time window (Fig. 3B). The second cluster (Fig. 3C) is related to the activation of immune system and complement pathways. Although this pattern also has a peak of gene expression at 3 months, the upregulation becomes much more pronounced at 9 months. Its Cnetplot shows that genes such as *C1qb*, C1qa, and *Trem2*, appeared as central nodes connecting multiple pathways and *Serping1*, *C4b*, *Cd68*, and *Tyrobp* shared this expression pattern (Fig. 3D). Gene Ontology enrichment analysis highlighted the significant terms including “humoral immune response”, “complement activation”, “lymphocyte-mediated immunity”, and “immunoglobulin-mediated immune response”. Additional enrichment in “synapse pruning”, “microglial cell activation”, and “regulation of complement activation” further supports the involvement of immune and complement-related processes. Box plot representing the expression of immune-related genes, including *C1qa*, *C4b*, *Trem2*, and *Cd68*, showed a significant upregulation at 3 months of age in *Prph2*^KI/WT^ mice compared with controls (Fig. 3E). To confirm the role of complement pathway, the percentage of retinal cells that express C3 was quantified using flow cytometry (Fig. 3F). In *Prph2*^KI/WT^ mice there was a significant increase of C3 positive cells at 3 months together with an increase of fluorescence intensity, which is consistent with the upregulation of genes involved in the complement cascade found in these mice compared with wild-type ones.

**Fig. 4:**
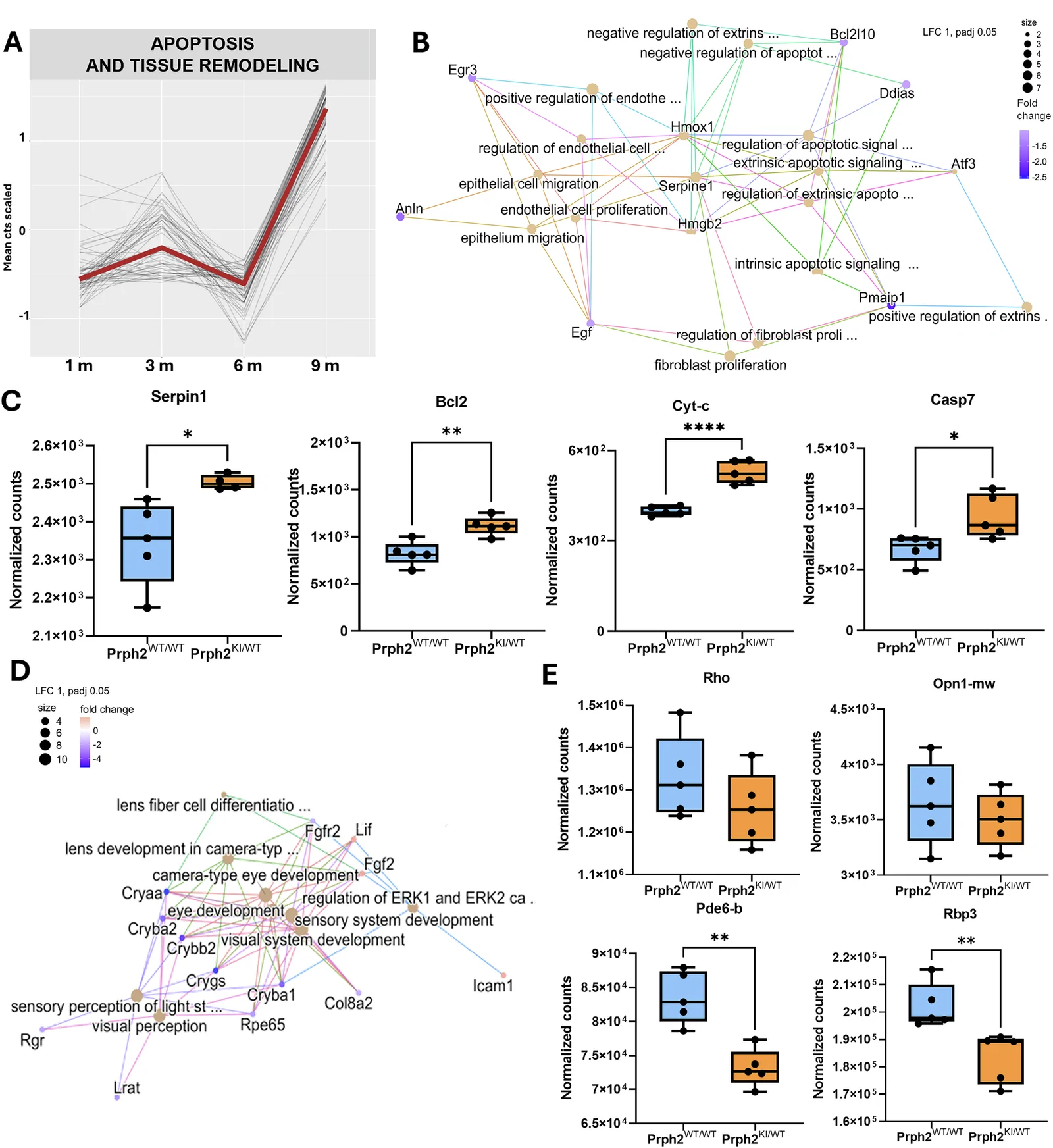
Age-dependent transcriptomic profiles of DEGs contributing to cellular turnover and neurodegenerative process. **A** Temporal expression profile of clustered genes contributing to cellular turnover displayed as a line plot. **B** Cnetplot illustrating DEGs involved in apoptosis and cellular remodeling at 6 months in *Prph2*^KI/WT^. **C** Box plot of normalized counts (TMM) of apoptotic markers overexpressed in *Prph2*^KI/WT^ compared to *Prph2*^WT/WT^ at 6 months of age. **D** Cnetplot of DEGs and related pathways to retinal tissue remodeling at 9 months old. **E** Box plot of normalized counts (TMM) of photoreceptors markers represented at 9 months of age in Prph2^KI/WT^ and *Prph2*^WT/WT^. All analyses were performed using the same set of animals with *n* = 20 per experimental group (*n* = 5 per age group, 4 age groups: 1, 3, 6, and 9 months). Values are expressed as mean ± SEM, *p-value ≤ 0.05; **p-value ≤ 0.01; ****p-value ≤ 0.0001.

Same analysis of DEGs was carried out at 6 and 9 months. However, no cluster could be established at 9 months because no genes shared the same expression profile. The Fig. 4A shows the genes identified at 6 months of age that are differentially expressed in *Prph2*^KI/WT^ mice compared with control mice and have same expression pattern. The cluster includes 58 genes that have a peak of expression at 9 months. The network analysis identified *Egf, Hmox1, Hmgb2*, and *Serpine1* as central hub genes, and additional genes, including *Pmaip1* and *Bcl2l10*, that formed multiple connections with distinct pathways. These genes are implicated in “regulation of apoptotic signaling”, “extrinsic apoptotic signaling”, and “intrinsic apoptotic signaling”, as well as in “epithelium migration” and “endothelial cell proliferation” (Fig. 4B). Moreover, the box plots represented in Fig. 4C show the significant upregulated genes (*Serpin1*, *Bcl2*, *Cyt-c*, and *Casp7*) in *Prph2*^KI/WT^ retinas at 6 months, that are involved in these cellular pathways. To complete the study at 9 months, the GO enrichment analysis of retinal DEGs in mutant mice compared with healthy ones (Fig. 4D). This analysis revealed strong interconnections between genes associated with “eye development”, “visual system development”, “camera-type eye development”, “lens development”, and “visual perception”. Central nodes, including *Cryaa*, *Cryba1/2*, *Crybb2*, and *Crygs*, formed a highly interconnected core. In addition, genes involved in “visual perception”, such as *Rgr*, *Lrat*, and *Rpe65*, were also dysregulated. Moreover, a reduced expression profile in visual cycle genes was observed. While *Rho* and *Opn1-mw* showed a decreasing trend, *Pde6b* and *Rbp3* were significantly downregulated, indicating impaired photoreceptor maintenance and function (Fig. 4E).

To determine whether the same pattern was observed in control mice, the temporal expression profiles of same gene clusters were represented in *Prph2*^WT/WT^ samples (Supplementary Fig. 1). The analysis reveals a gradual age-dependent trend with an expression pattern that differs from that observed in mutant mice. Moreover, substantially fewer genes contributed to the immune response, complement system, and apoptosis and tissue remodeling-related clusters.

### The characterization of sex-dependent signatures of inflammation reveals that retinal degeneration begins earlier in females than in males in *Prph2*^KI/WT^ mice

Our preliminary PCA of the transcriptomic data revealed a clear sex-based segregation of RNA expression profiles (Fig. 1D). This observation led us to further explore whether sex-dependent differences also contribute to the degenerative process. With this purpose, we redefined the groups based on the PCA results, corresponding to young (1 month), middle-aged (3 and 6 months), and old (9 months) stages. As it is possible to see in Supplementary Fig. 2, the PCA analyses performed separately in males and females reveal distinct clustering patterns across genotypes and stages of the disease. This analysis differed between males and females (males: PC1 = 15%, PC2 = 43%; females: PC1 = 17%, PC2 = 35%), suggesting sex-dependent differences in the relative contribution of age and genotype to the retinal transcriptomic profile.

**Fig. 5:**
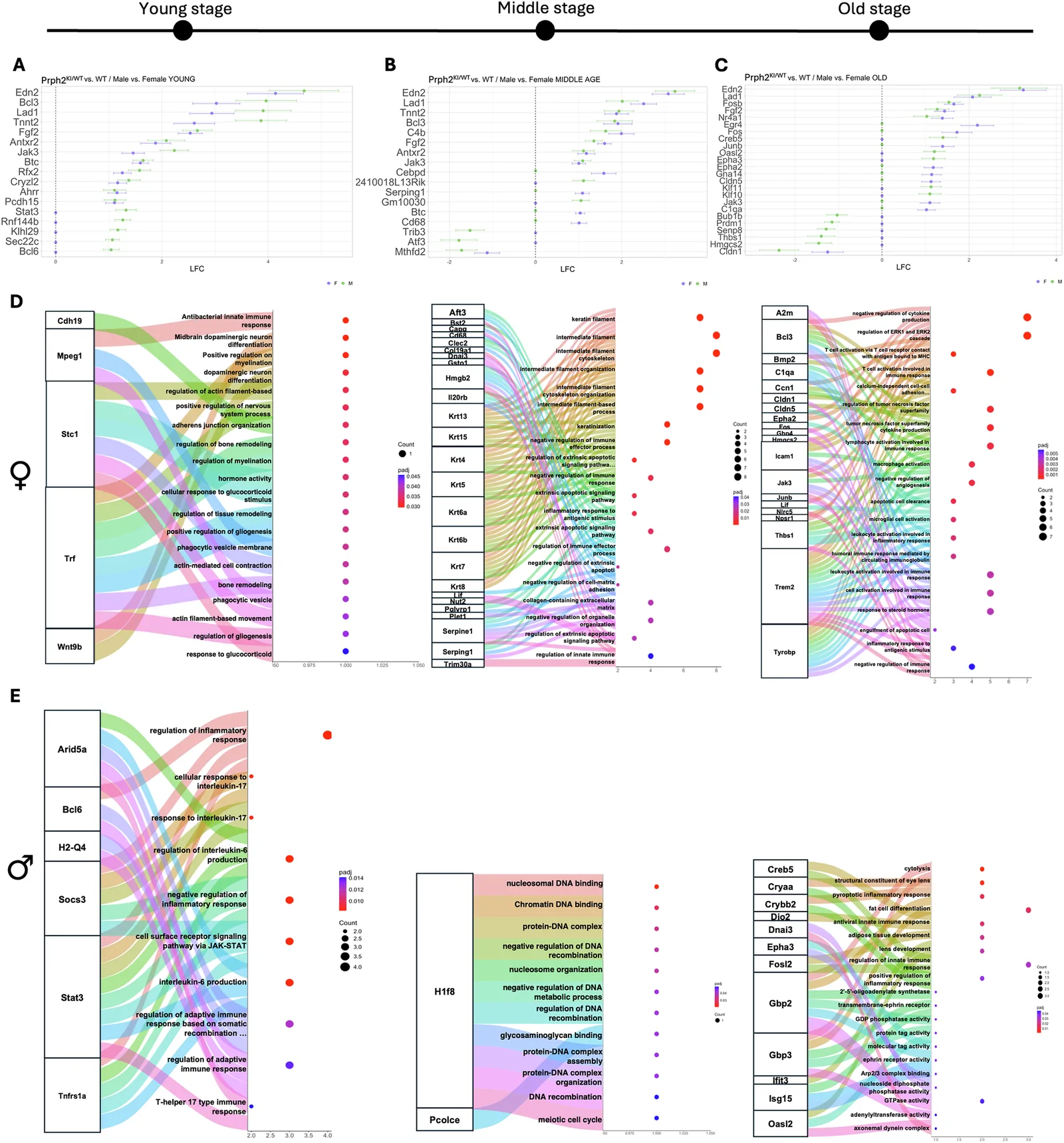
Sex-dependent deregulated DEG profiles and associated pathways across the retinal degeneration timeline. **A**–**C** Forest plots showing differential expression analysis with threshold (−1 < log2FC < 1) and *p*.adj < 0.05 between females and males in *Prph2*^KI/WT^ and *Prph2*^WT/WT^ at young stage (1 month), middle stage (3 and 6 months) and old stage (9 months) of retinal degeneration. Alluvial (left) and dot plots (right) of DEGs corresponding to biological functions in *Prph2*^KI/WT^ versus *Prph2*^WT/WT^ in females with (**D**) and males (**E**) along the 3 different stages, with threshold (−1 < log2FC < 1) and *p*.adj < 0.05. All analyses were performed using the same set of animals. At young and old stages of the disease, *n* = 2 males and 3 females in both *Prph2*^WT/WT^ and *Prph2*^KI/WT^ groups. At the middle-aged stage, *n* = 4 males and 6 females per experimental group.

Different forest plots were generated to visualize divergent transcriptional patterns between sexes at the three age stages in *Prph2*^KI/WT^ mice compared with *Prph2*^WT/WT^ mice (Fig. 5A–C). At young stage, 17 deregulated genes were identified and 8 of them (*Bcl3, Lad1, Tnnt2, Klhl29, Stat3, Rnf144b, Sec22c*, and *Bcl6)* showed increased expression (higher LFC) in males relative to females (Fig. 5A). When middle-age stage was analyzed, a list of 17 divergent genes was also identified. In this case, new genes appeared that were not present in the young stage group: *C4b, Cebpd, Serpin1, CD68, Trib3, Atf3*, the predicted gene *Gm10030*, and the long non-coding RNA (lncRNA) *2410018L13Rik*. Within these 17 genes, *Cebpd*, *Serping1*, *Btc*, *CD68, Trib3 and Atf3* display a significant increase in females compared with males (Fig. 5B). In contrast, *Gm10030 and 2410018L13Rik* showed increased expression in males compared with females. When comparing mice at the older age stage, 24 genes exhibited differential expression in the retina of mutant mice compared with controls. Moreover, 18 genes, out of 24 genes, exhibited a significant difference between sexes (Fig. 5C). Among the overexpressed genes, *Egr4, Fcs, Junb, Epha2, Gna14, Cldn5, Jak3*, and *C1qa* were significantly upregulated in females and *Creb5, Oasl2, Epha3, Klf11*, and *Klf10* in males. In addition, *Bulb1b, Prdm1, Senp8, Thbs1, Hmgcs2*, and *Cldn1* had decreased expression in old males compared with old females.

Based on this information and to further explore the biological pathways that could be influenced by these sex- and age-dependent gene expression patterns, results from mutant females and males were compared with their corresponding controls. Subsequently, genes that were differentially expressed exclusively in females or males were visualized with their representative pathways using an alluvial plot (Fig. 5E–D). At young age, this comparison points up the alterations of different pathways such as “positive regulation of myelination”, “dopaminergic neuron differentiation”, “phagocytic vesicle”, “regulation of actin filament-based movement” and “regulation of glycogenesis” in the retina of mutant females (Fig. 5D). In contrast, the *Prph2*^KI/WT^ males showed a variation in pathways like “regulation of inflammatory response”, “cellular response to interleukin 17” and “cell surface receptor signaling pathway via JAK-STAT” when compared with *Prph2*^WT/WT^ males (Fig. 5E).

When the same enrichment analysis was performed at middle-age stage (Fig. 5D), pathways such as “intermediate filament”, “extrinsic apoptotic signaling pathways” and “negative regulation of immune response” were found to be dysregulated in *Prph2*^KI/WT^ females compared to *Prph2*^WT/WT^ ones. On the other hand, “chromatin DNA binding” and “protein-DNA complex” were the unique altered pathways identified in the retina of mutant males (Fig. 5E). Finally, when *Prph2*^KI/WT^ mice are at older stages, an altered gene expression profile of “negative regulation of cytokine production”, “regulation of ERK1 and ERK2 cascade”, “macrophage activation”, “apoptotic cell clearance” and “microglial cell activation” pathways were found in females (Fig. 5D). In contrast, *Prph2*^KI/WT^ males presented disrupted signaling pathways like “cytolysis”, “pyroptotic inflammatory response”, “lens development” and “ephrin receptor activity” when compared with *Prph2*^WT/WT^ males (Fig. 5E), compared with control group.

**Fig. 6:**
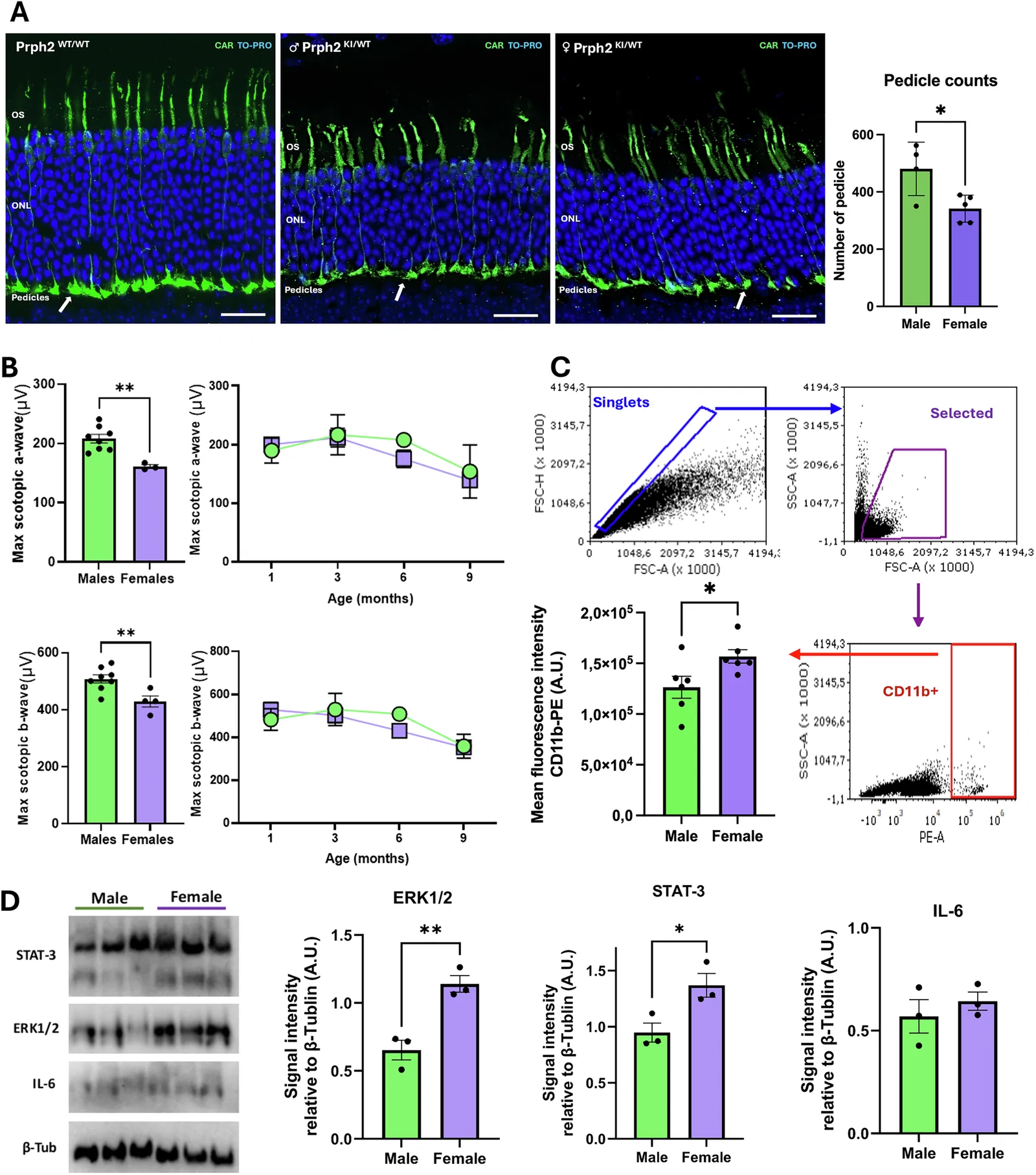
Exacerbated molecular, morphological and functional alterations during retinal degeneration in Prph2^KI/WT^ females. **A** Photoreceptor cells decrease in *Prph2*^KI/WT^ male compared with *Prph2*^KI/WT^ female at advanced stage of the disease (9–12 months). Cones were immunolabeled with cone-arrestin (green), and white arrow showing the pedicle counted. Bar graph showing decreased cone number in females (*n* = 5) compared with males (*n* = 4) in *Prph2*^KI/WT^ mice. Scale bars 20 µm. **B** Bar graph (left) showing the maximum amplitudes (µV) of the scotopic a-wave and b-wave recorded at the highest light intensity used (1 log cd s/m^2^) in ERGs in males (*n* = 8) and females (*n* = 4) at 6 months of age. Data are also represented as an aligned line plot (right) to facilitate the visualization of subtle changes and allow direct comparison between male and female results. **C** Flow cytometry gating strategy used to identify the CD11b⁺ population. Bar graphs show an increased immunoreactivity of Cd11b in females (*n* = 6) compared with males (*n* = 6) in *Prph2*^KI/WT^ animals at middle-aged stage. **D** Western Blot analysis showing increased levels of proteins associated with inflammatory signaling pathways in *Prph2*^KI/WT^ females (*n* = 3) compared with males (*n* = 3) in *Prph2*^KI/WT^ males at 3 months of age. Values are expressed as mean ± SEM. *p-value < 0.05 and **p-value < 0.01.

Given the sex-dependent transcriptomic differences observed in *Prph2*^KI/WT^ retinas, potential correlations with functional alterations were assessed. To study cone photoreceptors integrity, we examined retinal sections from male and female *Prph2*^WT/WT^ and *Prph2*^KI/WT^ mice at late stages of the disease, when clear differences of visual cycle genes where found. An immunostaining against cone arrestin (CAR, green) was performed and TO-PRO was used to stain the nuclei of the cells (blue). The Fig. 6A shows a representative picture of the immunostained cones in the retinas of *Prph2*^KI/WT^ male and female compared with wild-type one. When the number of cones pedicles was quantified, while there are no differences between sexes in control retinas (data not shown) a significant reduction was found in the retinas of *Prph2*^KI/WT^ females compared to *Prph2*^KI/WT^ males at same age (Fig. 6A).

To unravel potential sex-dependent differences in visual function, retinal responses to light stimuli recorded by ERGs were analyzed separately for males and females. The Fig. 6B shows the quantification of scotopic a- and b-wave ERG amplitudes and the representative wave traces used for quantitative analysis are shown in Supplementary Fig. 3. Results revealed a significant attenuation of retinal function in *Prph2*^KI/WT^ females compared to males, at middle-age stage of degeneration. Using the aligned line plot representation, it can be observed that the decline in the a- and b-wave response begins earlier in females than in males.

Following the observation that genes associated with the inflammatory pathways are more strongly dysregulated in *Prph2*^KI/WT^ female than in male, a comprehensive analysis of these processes was subsequently conducted. First, the percentage of microglia and macrophages (CD11b⁺ cells) was measured by flow cytometry. As is shown in Fig. 6C, although differences were not statistically significant a trend toward an elevated proportion of CD11b⁺ cells were observed, together with greater immunoreactivity in *Prph2*^KI/WT^ female compared to male retinas. Subsequently, protein levels of IL-6, STAT3, and ERK1/2 were evaluated by western blotting in *Prph2*^KI/WT^ at middle-age stage of degeneration. These three inflammatory biomarkers increased in females compared to male, reaching STAT3 and ERK1/2 statistical significance (Fig. 6D).

## Discussion

The present work aims to elucidate the pathophysiological mechanisms underlying the p.Arg195Leu mutation in the retina of *Prph2*^KI/WT^ mice. To our knowledge, this is the first study that analyzes the retinal transcriptome of an experimental model of CACD carrying the p.Arg195Leu mutation. Thus, the findings reported here are therefore both novel and highly relevant, providing critical insights into the molecular mechanisms that contribute to retinal degeneration specifically in CACD. Moreover, this work reveals that age and sex are two key factors that contribute to retinal degeneration [12], and can determine the onset and progression of CACD, as occurs in other pathological contexts such as age macular degeneration [13] or retinitis pigmentosa [14, 15].

Consistent with our previous results, these mutant mice experienced a reduction of retinal function along ageing [10]. Moreover, herein we demonstrate that from the early stages of the disease, visual impairment is accompanied not only with the disorganization of the OS of photoreceptors but also with the decrease of *Prph2* and *Rom-1* expression. At one month there is no photoreceptor cell death in these animals [10]. Considering that these two proteins have an important role in OS disc formation and maintenance [16], our results suggest that structural alterations in the OS compromise photoreceptor electrical function by itself in *Prph2*^KI/WT^ mice [11, 17]. As previously described, in these mutant mice, the structural alterations of the retina become more evident at advanced stages of the disease [10]. Accordingly, a clear sign of cones degeneration was observed in *Prph2*^KI/WT^ mice at 9 and 12 months of age, together with a marked downregulation of the essential pathways for vision, such as phototransduction and visual perception.

A major transcriptomic hallmark identified in *Prph2* mutant retinas along age is the robust upregulation of genes associated with the innate and adaptive immune response, including complement activation. From 1 month to 6, genes involved in the immune response, complement system and synapse pruning pathway were upregulated in retinas from *Prph2*^KI/WT^ mice when compared with control ones. This may reflect an early microglial activation, mediated by complement-dependent mechanisms [18].

From 3 to 6 months, the greatest differences in expression profiles are observed in retinas of mutant mice, as shown in the heatmap. Moreover, these molecular alterations are accompanied by concomitant cellular and functional changes. Thus, the middle-age represents a critical transition point at which the first signs of retinal degeneration start to emerge. At this stage, the overexpression of *Cd68*, could reflect the sustained activation of microglia and macrophages, consistent with an ongoing inflammatory response detected at 1 month in mutant retinas [19]. In line with this, the upregulation of *Trem2*, that was in the center of the network, also suggests the increase of microglial phagocytosis influencing the progression of photoreceptor degeneration [20]. We also detected overexpression of *Mpeg1*, a well-established marker of microglia and macrophages, further supporting their activation in our model [21]. In parallel, the upregulation of complement cascade genes, along with an increased percentage of cells expressing C3 protein in the retina of these mutant mice, demonstrate that complement cascade would potentially contribute to retinal degeneration. Moreover, when combined with the upregulation of apoptosis-related genes at these ages, these findings further support the idea that microglia activation is amplifying retinal damage caused by the mutation [19, 22–25]. In addition, activation of the complement pathway has been previously shown to involve Müller cells, which express the complement proteins following injury, thereby contributing to neuroinflammation and photoreceptor cell death [26].

Besides inflammation-related pathways, at the same age, genes involved in “epithelial differentiation” and “keratin and intermediate filament organization” were significantly deregulated in the retinas of *Prph2*^KI/WT^ mice. This data may indicate that the progressive epithelial barrier disruption at middle stages of CACD could compromise retinal integrity and promote retinal remodeling [27]. Also, additional genes related to fatty acids and retinoic acid metabolism become downregulated in the retina of mutant mice. These alterations, possibly linked to inflammatory processes triggered by the mutation [28, 29], suggest that with age retinal degeneration advances and affects key components of the visual cycle, including retinoic acid–related proteins. Moreover, retinoic acid receptors have been involved in the regulation of key homeostatic processes in the retina, particularly those related to lipid metabolism, oxidative stress, and inflammatory responses leading [30].

However, at this middle stage of the disease when mutant retinas were compared with healthy ones, not only a larger number of genes are deregulated, but also the variability between retinas of same experimental group was greater. As seen in humans, this could reflect the intra-familial variability, suggesting that genetic and environmental factors contribute to the differences observed among individuals of the same lineage [8, 9]. Based on our interpretation, this intra-group variability could be explained considering the sex-dependent differences observed in our results. Our transcriptomic stratification based on age and sex, as illustrated by PCA analysis, reveals a clear separation between early, intermediate, and late disease stages, with a clear distinction between male and female samples.

Notably, the functional, cellular and molecular alterations observed in *Prph2*^KI/WT^ mice do not affect males and females equally. This is further supported by the observation that mutant retinas from males exhibit an inflammatory profile only at younger ages (1 month) and develop a less severe disease course of CACD than females. In contrast with this, in females, a sustained inflammatory profile is evident, and from 3 months onward, this is associated with retinal degeneration. This early increase in inflammatory markers in *Prph2*^KI/WT^ mice may suggest that microglia adopts a dual phenotype, balancing neuroprotective and pro-inflammatory functions [31, 32]. In this line, the increase of CD11b⁺ population in the retinas of *Prph2*^KI/WT^ females at 3 months corroborates a pro-inflammatory environment [10, 33, 34], consistent with the expression of inflammation-related genes. As forest plots show, among the genes deregulated in mutant mice compared with wild-type ones, the retinas of *Prph2*^KI/WT^ females display an enhanced inflammatory profile, as evidenced in middle-age by the upregulation of *Cd68* and *C4b* [35]. The results from alluvial plots demonstrate that *Prph2*^KI/WT^ females display a higher proportion of DEGs in response to the mutation than *Prph2*^KI/WT^ males. These differences become more evident at middle-aged stage, where changes in the expression of keratin genes may indicate that in the retina of *Prph2*^KI/WT^ females, the tissue remodeling occurs earlier and involves RPE [36]. Even more, when retinal function was measured by ERG at same ages, *Prph2*^KI/WT^ females experienced a significant decrease in the a-wave. This may explain the detection of significant differences from the earliest stages of disease in the present study, in contrast to our previous work [10]. The use of a balanced number of male and female animals may underlie these differences, as female animals appear to exhibit a more severe phenotype in this model, which may have influenced the results. The integration of the RNA-seq data with the western blot results suggests that the activation of the IL‑6-mediated STAT3/ERK signaling pathway may contribute to the more pronounced retinal inflammation and tissue remodeling observed in *Prph2*^KI/WT^ females [37, 38].

## Conclusion

Our findings indicate that inflammation and specifically complement cascade, could be considered the main contributor of retinal degeneration in CACD. This work clearly suggests that the onset of CACD occurs earlier in females than males and the retinal degeneration progresses differently depending on the sex, being more severe in females. It is essential to consider sex as a biological variable when developing therapeutic strategies for this mutation and for those that compromise the D2 loop of PRPH2. These new insights highlight that accounting for these age- and sex-specific differences would be necessary for developing personalized treatments and improving their efficacy in retinal degenerative disease.

## Materials and methods

### Animals

C57BL/6J-Prph2em1Sal mouse model carrying the p.Arg195Leu mutation was used to study the CACD pathology. This knock-in model was generated by CRISPR/Cas technology mimicking the human disease as previously described in Ruiz-Pastor et al. [10].

Wild-type (*Prph2*^*WT/WT*^) and *Prph2*^KI/WT^ mice were bred in the animal facility of the University of Alicante and maintained under controlled temperature (23 ± 1 °C), humidity (55–60%), photoperiod (12/12 h light/dark), and light intensity (300 lux during the light period). Food and water were provided *ad libitum*. We always ensured a randomized distribution of the animals in each age group- 1, 3, 6 and 9 months of age- and an equal number of females and males for each experiment. All animal procedures were approved by the Ethical Committee of the University of Alicante (2023/VSC/PEA/0280) and performed following EU Directive 2010/63/EU. For all procedures where anesthetization was required, mice were anesthetized with 100 mg/kg ketamine and 10 mg/kg xylazine by intraperitoneal injection. Animals were always euthanized by cervical dislocation.

### Electroretinography recordings

Electroretinogram (ERG) responses were recorded under complete darkness following overnight dark adaptation at 1, 3, 6, and 9 months of age in *Prph2*^WT/WT^ (*n* = 25) and *Prph2*^KI/WT^ (*n* = 25) animals, using the HMsERG LAB System (OcuScience, Henderson, NV, USA). Animals were anesthetized with Tropicamide 1% (Alcon Cusí, Barcelona, Spain) applied topically was used for pupil dilation. Moreover, to prevent eye dehydration and ensure electrical contact with the recording electrodes, a 0.2% polyacrylic acid carbomer Viscotears (Novartis, Barcelona, Spain) was employed during the procedure. Retinal responses were recorded bilaterally. Scotopic potentials were evoked by using 14 light stimuli with increasing luminance (−5.5 to 1logcd s/m^2^). The amplitude of the a-wave was quantified as the distance from baseline to the most negative trough, whereas the b-wave amplitude was measured from the a-wave trough to the maximum peak of the b-wave. Data are presented as the maximum recorded amplitude (µV) of the scotopic a-wave and b-wave recorded at the highest light intensity used (1 logcd s/m^2^).

### Immunofluorescence

After euthanizing the animal, the dorsal margin of the limbus was marked by a suture. The eyes were enucleated and fixed at room temperature for 1 h using 4% paraformaldehyde (PFA). After 3 washes with 0.1 M phosphate buffer (PB; pH 7.4), solutions of increasing sucrose concentrations (15–20–30%) were employed to cryoprotect the tissue. Sucrose at 15% and 20% was applied for 1 h, and 30% of sucrose was maintained overnight at 4 °C. The cornea, lens, and vitreous body were removed, and the eyecups were embedded in Tissue-Tek OCT compound (Sakura Finetech, Tokyo, Japan) using liquid nitrogen. Sections of 15 µm thickness were obtained with a cryostat (CM1950; Leica Microsystems, Wetzlar, Germany), collected on Superfrost glass slides (J1810AMNZ, Thermo Fisher Scientific, Whaltham, MA, USA) and stored at −20 °C. For the immunostaining procedure, sections were thawed, washed in PB, and incubated at room temperature for 1 h in blocking solution consisting of 10% (v/v).

Donkey serum (S30 Merk Millipore, Darmstadt, Germany) in PB with 0.5% Triton X-100 (Sigma-Aldrich, St. Louis, MO, USA). Right after, sections were incubated overnight at 4 °C with rabbit anti-cone arrestin (1:200, AB15282, Millipore) or rabbit anti-Prph-2 (1:1000), 18109-1-AP, ProteinTech, Rosemont, Illinois, USA) primary antibodies diluted in blocking solution. Slides were then washed in PB and incubated with the secondary antibody donkey anti-rabbit Alexa-488 (1:100, A21206, Molecular Probes, Eugene, OR, USA) for 1.5 h. To stain the nuclei, upon PB washings, TO-PRO-3 iodide dye (R37113, Invitrogen, Carlsbad, CA, USA) was incubated for 20 min. Finally, sections were washed in PB and mounted with Fluoromount (00495802, Invitrogen) under a coverslip.

Non-consecutive whole section images from each animal were taken with a Leica TCS SP8 confocal laser-scanning microscope (Leica Microsystems, Wetzlar, Germany) using a 40× objective. For quantifications, Photoshop software (Adobe) was employed and at least two non-consecutive images from the whole retina of each mouse were used. The arrestin-positive pedicles of cones were counted manually in the whole retina, and an average number was calculated.

### Transcriptome sequencing and bioinformatic analysis

Mice were euthanized by cervical dislocation. After enucleation, neural retinas were carefully dissected by removing the anterior segment, vitreous body, sclera and detaching them from the RPE. Retinas were then washed with PBS to remove residual RPE cells. Upon dissection, neural retinas were immediately frozen in liquid nitrogen and stored at −80 °C until use. Total mRNA from control *Prph2*^WT/WT^ mice (*n* = 25) and *Prph2*^KI/WT^ mice model (*n* = 25) at 1, 3, 6 and 9 months (*n* ≥ 5 for each group) was isolated from the fresh-frozen retina using QiAzol Lysis Reagent (Qiagen, 79306) followed by RNeasy Micro Kit (cat. No.74004) according to the manufacturer’s protocol. RNA concentrations and the ratio 260/280 were assessed using the NanoDrop One C (Thermo Fisher Scientific). Moreover, before carrying out the RNA-Seq analysis, RNA samples passed quality control based on RQN values (RNA Quality Number) and concentration thresholds defined by the company Genewiz from Azenta Life Sciences (Germany). Only RNA sample with RQN > 9 were processed.

Library preparation was assessed with NEBNext Small RNA Library Prep Set for Illumina, by the company GENEWIZ AZENTA. The mRNA transcriptome was profiled using bulk-RNA sequencing Illumina NovaSeq (150 bp paired-end), 30 million reads per samples by the company GENEWIZ AZENTA. Pipeline post-sequencing QC, trimming, alignment and mapping were assessed by the company GENEWIZ AZENTA. Raw count data comprising 49,315 genes were first filtered to remove low-quality or non-informative features. Genes with a total count of zero across all samples (38,585 variables) or with counts below 60 in more than three samples (14,755 variables) were excluded. Data normalization and differential expression analysis were performed using the DESeq2 package [39] (v1.46.0) in R (v4.4.2). Differentially expressed genes (DEGs) were defined as those with an adjusted *p*-value (Benjamini–Hochberg correction) ≤0.05 and an absolute log2 fold change ≥1.

Functional enrichment of DEGs was performed using the clusterProfiler [40–42] package (v4.16.6). Both Gene Ontology (GO) terms (Biological Process, Cellular Component, and Molecular Function) and KEGG pathway analyses were conducted. Overrepresentation analysis was performed using a hypergeometric test, and *p*-values were adjusted for multiple testing using the Benjamini–Hochberg method. Only terms with adjusted *p*-value ≤ 0.05 were considered significantly enriched. Visualization of enriched terms was generated using dot plots and enrichment maps implemented in clusterProfiler. Hierarchical clustering was performed on normalized and variance-stabilized count data obtained from DESeq2. Expression values were first log-transformed, transposed, and scaled (Z-score normalization across samples) to emphasize relative expression dynamics between genes. A distance matrix was computed using the Euclidean distance, and hierarchical clustering was performed with the Ward’s minimum variance method (ward.D2) using the hclust function from the *stats* package in R (v4.4.2).

Line plots of temporal expression profiles and cnetplots were generated using normalized counts of *Prph2*^KI/WT^ DEGs compared to WT samples, in order to highlight temporal trends of disease progression.

### Flow cytometry

Cell populations related inflammation were studied by flow cytometry in retinas from *Prph2*^WT/WT^ and *Prph2*^KI/WT^ animals at 1, 3 and 6 months of age. Following euthanasia, the eyes were enucleated, and the retinas were dissected and disaggregated in 1 ml of PBS using a wide-bore pipette tip. To remove cell aggregates, the suspension was filtered through a 30 µm strainer (BD Biosciences, San Diego, CA, USA). Fixation and permeabilization was conducted by using Fix & Perm™ reagent kit (GAS004, Thermo Fisher Scientific) following the manufacturer’s recommendations. Samples were labeled with anti-CD11b-PE antibody (Clone M/170, e-Bioscience), anti C3 (Hycult, Uden Netherlands), and the secondary antibody anti-rat FITC (Jackson, West Grove, PA, USA). Each mouse retina was analyzed individually and at least 4 animals were included in each experimental group. Data were acquired on a Cytek Aurora Spectralis cytometer (Cytek Biosciences, Fremont, CA, USA) and analyzed using FCS 7 Flow Cytometry Software (De Novo, Los Angeles, CA, USA).

### Western blotting

Protein levels were analyzed by SDS-Page. Neural retinas were lysed with RIPA buffer (R0278, Sigma-Aldrich, Germany), PhosSTOP (04906837001, Roche, Germany), and protease inhibitor cocktail complete Mini EDTA-free (04693159001, Roche). Protein quantification was carried out using the Bradford assay (Sigma), with bovine serum albumin (BSA) used as standard. Equal protein concentrations from each sample (20 μg) were loaded onto 4–12% SDS-polyacrylamide gels and subsequently transferred to polyvinylidene difluoride membranes (PVDF) (IPFL10100. Merk Millipore) by wet transfer. Membranes were incubated overnight with primary antibodies anti-STAT-3 at (1:1000, Cell Signaling, 9139S), anti-ERK ½ (1: 1000, Cell Signaling, 9102S) and anti-IL-6 (1: 250, Santa Cruz Biotechnology, sc-1265) and secondary antibodies (1:1000, Invitrogen, 32430) were incubated afterwards at room temperature for 2 h. Anti-β-tubulin (1:500, Santa Cruz Biotechnology, sc-5274. Bands were obtained using Pierce^TM^ ECL Plus (32132, thermo scientific, Rockford, IL, USA) on Molecular Imager Chemidoc^TM^ XRS+ (Biorad). Full and uncropped western blot images are shown in Supplementary Material Fig. 4. ImageJ software (National Institutes of Health [NIH], Bethesda, MD, USA) was used to quantify protein levels by densitometry. The results were normalized using the housekeeping protein and represented as a percentage relative to the control group.

### Statistical analysis

No statistical methods were used to predetermine sample size. Sample sizes were selected based on previous studies using similar experimental models and transcriptomic approaches, as well as our prior experience with the model [10]. The number of animals was chosen to provide sufficient biological replication while adhering to the principles of animal welfare and reduction. No randomization was used. Animals were assigned to groups based on genotype and age. No blinding was performed during the experiments or data analysis.

Normalized gene expression counts obtained from bulk RNA-seq were analyzed using R (v4.4.2). Boxplots were generated with the ggplot2 package to visualize gene expression across experimental groups, with individual data points overlaid using geom_jitter to show sample distribution. Group comparisons (e.g., male vs. female within each genotype) were performed using unpaired two-tailed Student’s *t* tests via the stat_compare_means function from the ggpubr package. Data are presented as boxplots indicating the median, interquartile range (IQR), and individual observations. A significant threshold of *p* < 0.05 was applied.

Statistical analysis of electroretinogram, immunofluorescence, FACs and western blot data was performed using GraphPad Prism Analysis Software (GraphPad Software, Inc., San Diego, CA, USA). Outliers were identified and excluded using ROUT and Grubbs’ test. Kolmogorov–Smirnov or Shapiro–Wilk tests were used in each experiment to assess the normality, and homoscedasticity was assumed. ANOVA and Tukey’s post hoc test Kruskal–Wallis test was applied to determine the significant differences among groups. An unpaired *t*-test or the non-parametric Mann–Whitney test was used for comparisons between two groups. Data are presented as the mean value ± SEM and *P* < 0.05 was considered statistically significant.

The RNA-seq data generated in this study will be deposited in a public repository upon completion of the analysis and will be made available upon publication.

## Supplementary information

Supplementary figure legends

Supplementary Figure 1

Supplementary Figure 2

Supplementary Figure 3

Supplementary Figure 4

## Data Availability

The datasets generated during and/or analyzed during the current study are available from the corresponding author on reasonable request.
