# Supplementary material for: Age- and sex-dependent retinal degeneration in peripherin-2 mutant mice, a model of central areolar choroidal dystrophy
Source: Cell Death Discov. 2026 Jul 23;12:419. doi: 10.1038/s41420-026-03263-z (PMC13680364; doi:10.1038/s41420-026-03263-z)
Supplement: Supplementary file 1 [file 41420_2026_3263_MOESM1_ESM.docx]

# Supplementary Figure legends

**Supplementary Figure 1:** Line plot of the temporal expression profiles gene clusters in Prph2^WT/WT^ samples, associated with **(A)** epithelial barrier function, **(B)** immune response and complement system, and **(C)** apoptosis and tissue remodeling.

**Supplementary Figure 2: Gene expression–based clustering reveals sex- and age-dependent separation. (A)** Principal Component Analysis (PCA) of RNA-seq data illustrating the variance in global gene expression profiles between experimental groups and across age categories, after grouping samples into young (1 month), middle-aged (3 to 6 months), and old (9 months) categories. Samples are additionally separated by sex (female, top; male, bottom). Note the distinct clustering of male and female samples across both experimental groups, regardless of age.PC1 and PC2 explain 28% and 26% of the total variance, respectively. **(B)** PCA of same data set illustrating the variance in global gene expression profiles in male and female **(C)** clustering the retinal transcriptomic profiles across genotypes and different stages of the disease. At young and old stages of the disease, n = 2 males and 3 females in both Prph2^WT/WT^ and Prph2^KI/WT^ groups. At the middle-aged stage, n = 4 males and 6 females per experimental group

**Supplementary Figure 3:** Representative scotopic ERG waveforms obtained from Prph2^KI/WT^ male (left, green) and female (right, purple) mice at 6-month age, illustrating the mixed rod-cone responses measured under dark-adapted conditions.

**Supplementary Figure 4:** Full and uncropped western blot images used to analyze (A)ERK1/2, (B)IL6, and (C)STAT3 protein levels. Densitometric values were normalized to β-tubulin (D). Each lane represents one retina (3 female and 3 male) from Prph2^KI/WT^ mice at 3 months of age.
